# Diseases of Stomach and Intestines

**Published:** 1905-12-09

**Authors:** 


### DISEASES OF STOMACH AND INTESTINES.

**Dilatation of the Stomach.**—The determination of this condition by physical signs has been the object of various more or less elaborate procedures. The stomach has been filled with fluorescine and examined by means of internal illumination, and the *x*-rays are also employed. Benedict [1] has recently described a system of auscultatory percussion which he says gives good and reliable results in most cases. It merely consists in ordinary percussion or the use of a tuning-fork or some special percussor, the sounds being listened to through a binaural stethoscope. This method, at any rate, has the advantage of simplicity, and it may be doubted if the more elaborate ones are ever really necessary to establish a diagnosis. Einhorn [2] terms clinical dilatation ischochymia, which appears quite unnecessary. He points out that many cases due to benign stenosis of the pylorus get quite well with medical treatment. Those which are obstinate, or where there is a suspicion of malignancy, should be operated upon. Many other physicians speaking at the American Gastro-enterological Association agreed in this view, but surgeons generally considered that operation was needed in all cases of pyloric obstruction great enough to cause dilatation and not dependent on syphilitic ulceration near the pylorus. In this country a similar difference of opinion prevails. Elsner [3] has classified the causes of benign stenosis into congenital and acquired. The former may be complete or incomplete; the latter functional (transitory) or organic. Organic causes are (1) Intrapyloric—namely, chronic cicatricial stenosis; stenosis following ulcer; acute inflammatory stenosis associated with infection; deep-seated gastritis and acute poisoning; syphilitic stenosis; tuberculous stenosis; stenosis from non-malignant growths, distortion, or traction. (2) Extrapyloric from various sources of pressure. Dilatation of the stomach has been noted to give rise occasionally to tetany. Trimble [4] has reported such a case in a man aged 45, who had suffered from symptoms of considerable dilatation for years. He had a series of three well-marked attacks with carpopedal contractions, but without loss of consciousness. He became incoherent, and finally comatose, and died on the seventh day, having suffered from severe hiccough throughout the illness. Trimble regards the mental condition and hiccough as unusual. He states that tetany following gastric dilatation is rare, and nearly always fatal. The two theories as to its causation are that it is a toxæmia originating in the alimentary canal, or a reflex nervous irritation. He thinks that these combined would satisfactorily account for the symptoms. Toxic material being absorbed acts especially on the delicate nerve-endings of the hands and feet, producing reflex convulsions; it also in severe cases sets up inflammatory changes in the anterior cornua. He recommends Mayo Robson's plan of performing gastroenterostomy as likely to give the best results if the patient survives an attack.

**Gastric Erosions.** — These have recently been discussed by Funke,[5] who describes a case of parenchymatous nephritis, in which numerous erosions were found post-mortem. He does not differ from the descriptions given by other writers, but attributes the erosions to the excretion of poisons into the stomach, such as occurs with metallic poisons into the colon. The ulcers he thinks similar to those of stomatitis or colitis.

**Appendicitis.**—The causative factors in an attack of appendicitis have been summarised by Bottomley,[6] who divides his subject into three parts: First, as to the bacteria concerned, he finds the colon bacillus most frequently present either alone or with the streptococcus pyogenes. The latter may also occur alone, in which case the pus will have no offensive odour. Staphylococci, pneumococci, putrefactive bacilli, and others are occasionally found. Tubercle, actinomyosis, and typhoid ulcers occasionally affect the appendix. All attacks of appendicitis in the ordinary sense are probably due to bacteria, the virulence of which, together with the resisting powers of the patient, determines the severity of the attack. Secondly, Bottomley reviews the reasons which render the appendix particularly liable to attacks of inflammation. First among these he places the presence of abundant lymphoid tissue in the appendix and the large numbers of bacteria normally found in the cæcum. In some cases he considers these two conditions are enough to account for recurrent attacks; in other cases there is some previous morbid condition in the appendix, such as concretions, ulcers, or stenosis. The usual sequence of events appears to him to be chronic catarrh leading to either concretions or stenosis, and sometimes to both, or to ulceration, which, too, may accompany either. The organisms are thus encouraged to grow and develop toxins, which produce an acute attack. True foreign bodies are rare. Kinking or twisting of the appendix sometimes gives rise to stenosis and a consequent acute appendicitis. Thirdly, the general conditions giving rise to chronic catarrh are dealt with. Bottomley believes that there is an actual increase in the number of cases of the disease, and, seeing that appendicitis is much commoner in men than in women, is not disposed to credit constipation and the taking of purgatives as important causes. The bolting of indigestible masses of food he thinks more important, and with this classes chronic indigestion, bad teeth, and hurried meals as important predisposing circumstances. The uric-acid diathesis he does not think of special moment as a producer of appendicitis. He attributes the frequency of the disease in children to bad teeth, improper food, and the chronic intestinal indigestion to which many children are subject.

**Spastic Constipation.**—Atonic conditions of the musculature of the intestine are not the only causes of constipation. A form known as spastic constipation has been described, and is dealt with by Albu.[7] It apparently consists of spasmodic contractions of the gut supervening on chronic atony, and is commoner in neurasthenic and weakly females between thirty and fifty. Other muscular spasms may accompany it, and the attacks vary in length from hours to weeks, with intervals of merely atonic constipation. A tumour is occasionally felt, but Albu admits that the diagnosis is often difficult and sometimes impossible. If it should be made with any degree of certainty, the treatment is hot baths and compresses with large oil enemata and atropine suppositories. Vegetable food freed from husks and shells and other forms of cellulose and cereal gruels are recommended, with moderate exercise in the open air. It seems probable that the diagnosis may have to be made upon the result of treatment, which is never a very satisfactory proceeding.

**Dysentery.**—The South African war, as did the Spanish-American war in the United States, has led to a large number of communications on this subject. Faichnie,[8] who divides the disease in the usual way into endemic or amœbic, sporadic (due to mechanical irritation from food), and epidemic or bacillary, mainly deals with the latter as seen in South Africa. The greater part of his paper deals with treatment, and may be summarised as follows:—Prevention is summed up in early notification, isolation, and disinfection of latrines and infected persons. Mild cases may be treated on the spot and soon recover; severe cases should be sent to the base hospitals. Rest and equable temperature are essential, with sulphate of magnesia in the acute cases—drachm doses every hour till the stools become fæcal, and then at longer intervals. Perchloride of mercury and tr. chloroformi et morphinæ should be given in the later stages and bismuth or sulphur with opium to stop the diarrhœa. In chronic cases magnesium sulphate is useless, and local treatment with bicarbonate of soda and iodine must be adopted. As to diet, milk is only suitable for mild cases; albumen water or whey should be substituted in bad ones. When neither fresh milk nor eggs are to be got, whey may be made from tinned milk by precipitation with alum, or plasmon may be used. Transfusion is most valuable for collapsed conditions, and digitalis when the pulse is very weak is far better than alcohol. Opinions as to the value of ipecacuanha seem to differ considerably. Faichnie does not think it of much value; but Buchanan,[9] also as a result of South African experience, states that it does good where sulphate of magnesia fails. Dealing with the mild cases observed in America, Park[10] states that in these

Shiga's bacillus is not found, but a group of allied organisms known as paradysenteric bacilli are very frequently present, and are also found in cases of cholera infantum. In temperate climates the disease is so mild that serum treatment is unnecessary, a view in which Huddleston agrees. The latter points out that the worst cases often occur in the middle of winter, and should be treated with saline enemata, with salol and castor oil by the mouth. Morphia should be given when necessary.

[1] Med. Rec., June 17, 1905, p. 954. [2] Ibid. p. 953. [3] Ibid., p. 955. [4] B. M. J., May 5, 1905, p. 986. [5] Lancet, May 20, 1905, p. 1372. [6] Pract., June, 1905, p. 827. [7] Med. Rec., July 1, 1905, p. 14. [8] B. M. J., August 12, 1905, p. 325. [9] Ibid., p. 326. [10] Med. Rec. loc. cit., p. 955.

